# Blackwater fever and acute kidney injury in children hospitalized with an acute febrile illness: pathophysiology and prognostic significance

**DOI:** 10.1186/s12916-022-02410-4

**Published:** 2022-07-01

**Authors:** Andrea L. Conroy, Michael T. Hawkes, Aleksandra Leligdowicz, Ivan Mufumba, Michelle C. Starr, Kathleen Zhong, Sophie Namasopo, Chandy C. John, Robert O. Opoka, Kevin C. Kain

**Affiliations:** 1 Department of Pediatrics, Indiana University School of Medicine, 1044 West Walnut St., Building 4, Indianapolis, IN 46202 USA; 2 Division of Pediatric Infectious Diseases, 3-593 Edmonton Clinic Health Academy, University of Alberta, Edmonton, AB T6G1C9 Canada; 3 Division of Critical Care Medicine, Robarts Research Institute, University of Western Ontario, 1511 Richmond St, London, ON N6A 3K7 Canada; 4 Global Health Uganda, Kampala, Uganda; 5 Sandra Rotman Centre for Global Health, Toronto General Hospital, University Health Network and University of Toronto, Toronto, ON M5G1L7 Canada; 6 Kabale District Hospital, Kabale, Uganda; 7 Department of Paediatrics and Child Health, Makerere University, Kampala, Uganda

**Keywords:** Acute kidney injury, Malaria, Blackwater fever, Mortality, Children

## Abstract

**Background:**

Acute kidney injury (AKI) and blackwater fever (BWF) are related but distinct renal complications of acute febrile illness in East Africa. The pathogenesis and prognostic significance of BWF and AKI are not well understood.

**Methods:**

A prospective observational cohort study was conducted to evaluate the association between BWF and AKI in children hospitalized with an acute febrile illness. Secondary objectives were to examine the association of AKI and BWF with (i) host response biomarkers and (ii) mortality. AKI was defined using the Kidney Disease: Improving Global Outcomes criteria and BWF was based on parental report of tea-colored urine. Host markers of immune and endothelial activation were quantified on admission plasma samples. The relationships between BWF and AKI and clinical and biologic factors were evaluated using multivariable regression.

**Results:**

We evaluated BWF and AKI in 999 children with acute febrile illness (mean age 1.7 years (standard deviation 1.06), 55.7% male). At enrollment, 8.2% of children had a history of BWF, 49.5% had AKI, and 11.1% had severe AKI. A history of BWF was independently associated with 2.18-fold increased odds of AKI (95% CI 1.15 to 4.16). When examining host response, severe AKI was associated with increased immune and endothelial activation (increased CHI3L1, sTNFR1, sTREM-1, IL-8, Angpt-2, sFlt-1) while BWF was predominantly associated with endothelial activation (increased Angpt-2 and sFlt-1, decreased Angpt-1). The presence of severe AKI, not BWF, was associated with increased risk of in-hospital death (RR, 2.17 95% CI 1.01 to 4.64) adjusting for age, sex, and disease severity.

**Conclusions:**

BWF is associated with severe AKI in children hospitalized with a severe febrile illness. Increased awareness of AKI in the setting of BWF, and improved access to AKI diagnostics, is needed to reduce disease progression and in-hospital mortality in this high-risk group of children through early implementation of kidney-protective measures.

## Background

Acute kidney injury (AKI) is common, under-recognized, and associated with increased mortality [1]. An estimated 85% of AKI cases occur in low-and-middle-income countries (LMIC) [2]. In contrast to AKI in high-income settings, AKI in LMICs is more likely to be community-acquired and often occurs during infection in previously healthy children [3]. A lack of awareness of the importance and diagnosis of AKI and insufficient access to creatinine-based diagnostics contribute to ongoing under-recognition and under-reporting of AKI in LMIC settings [3]. AKI is an established risk factor for chronic kidney disease (CKD) [4, 5] and new disabilities at hospital discharge in both high-income and LMIC settings [5–7]. Furthermore, among patients hospitalized with severe malaria, the presence of AKI is associated with an increased risk of neurocognitive and behavioral complications that persist after hospital discharge [5, 8].

Malaria and sepsis are leading causes of AKI in children in sub-Saharan Africa [9]. Recent estimates suggest that AKI occurs in 24–59% of children with severe malaria [10]. An increase in AKI recognition in severe malaria has coincided with increased reports of blackwater fever (BWF) in African children, particularly in Eastern Uganda and the Democratic Republic of Congo [11]. BWF is a complication of severe malaria in which hemolysis predominates. Intravascular hemolysis results in an accumulation of hemoproteins that can overwhelm the capacity of endogenous hemoprotein scavengers (e.g., haptoglobin, hemopexin) [12–15]. The kidney proximal tubular epithelium is the primary route of hemoprotein clearance when endogenous scavenger systems are saturated [15]. Exposure of renal proximal tubular cells to excess cell-free hemoglobin and heme can lead to direct cellular injury and AKI through increased oxidative stress, tubulointerstitial inflammation, and endothelial activation [15]. BWF is a risk factor for hospital readmission and post-discharge mortality in African children [16, 17]. However, the relationships between AKI and BWF have not been systematically investigated.

In the present study, we evaluated the association between AKI and BWF in a prospective cohort of Ugandan children hospitalized with an acute febrile illness. As secondary objectives, we quantified circulating markers of immune and endothelial activation to define biologic mechanisms underpinning AKI and BWF. Finally, we examined the association of AKI and BWF with subsequent mortality.

## Methods

### Study population

This study was nested within a previously described prospective cohort study of children hospitalized with an acute febrile illness at Jinja Regional Referral Hospital in Uganda [18, 19]. The study was registered on clinicaltrials.gov (identifier: NCT04726826). Jinja Regional Referral Hospital is a referral hospital for the Busoga sub-region of Uganda comprising 8 districts and serves a catchment population of nearly two million people. Jinja is located 81 km east of the capital city, Kampala, on the shores of Lake Victoria. Jinja has one of the highest rates of severe anemia in the country and malaria is a frequent cause of anemia. Children were consecutively recruited between February 15, 2012, and August 29, 2013. Inclusion criteria were age 2 months to 5 years; parental report of fever within the past 48 h, or axillary temperature greater than 37.5 °C; hospitalization according to the admitting physician’s judgment; and parent/guardian consent to blood sampling and data collection. Exclusion criteria included diarrheal illness without other symptoms. Participant enrolment occurred between 08:00 and 20:00 with children presenting after 20:00 having a sample collected the following day. Of the 2502 children enrolled in the study, only 11 presented to the hospital after working hours and had a blood sample collected the following day.

### Study design

The present analysis was conducted as a sub-study nested within a previously described prospective cohort study [18, 19]. Our primary objective was to define the association between BWF and AKI among children admitted with febrile illness in Uganda. As secondary objectives, we evaluated the association between BWF and AKI, and (i) host response, and (ii) mortality. A standard sample size calculation indicated that we would need a minimum 915 patients to detect a statistically significant association between BWF and AKI at admission, assuming a prevalence at admission of both conditions of 10%, and an odds ratio of 1.4 or greater, at the *α* = 0.05 level of confidence, with 90% power. Based on this, a random subset of 1000 study participants from the parent study [18] were selected for the current analysis using the sample function in STATA.

### Study procedures

On enrollment, all children had a history and physical examination and a single venous blood draw. The blood sample was collected into a BD Microtainer® blood collection tube with EDTA (Becton, Dickinson and Company) and processed within 4 h. Microtainer tubes were centrifuged for 20 min at 1360 × g and the plasma layer collected and stored in a cryovial at 4 °C until transport from Jinja, Uganda, to Kampala, Uganda. Samples were transported to Kampala daily on ice and transferred to a − 80 °C freezer within 24 h of sample collection. Cryopreserved samples were shipped to Toronto, Canada, on dry ice, where the cryovials were stored at − 80 °C without freeze–thaw until batch analyte quantification. Malaria was assessed using a three-band rapid diagnostic test (RDT) with *Plasmodium falciparum* histidine-rich protein 2 (HRP2) and pan-malaria lactate dehydrogenase (pLDH) (First Response Malaria Ag. HRP2/pLDH Combo Rapid Diagnostic Test, Premier Medical Corporation Limited, India) [20]. Blackwater fever (BWF) was defined as parental report of tea or “Coca-Cola”-colored urine on enrollment which has a reported agreement of 80% with urine dipsticks positive for hemoglobin[5].

### Assessing kidney function

AKI was defined using the Kidney Disease: Improving Global Outcomes (KDIGO) criteria based on a 1.5-fold increase in serum creatinine on enrollment from the estimated baseline. AKI was staged as follows: stage 1, 1.5–1.9-fold increase in creatinine over baseline; stage 2, 2.0–2.9-fold increase over baseline; and stage 3, ≥ 3.0-fold increase over baseline. AKI was classified as severe (severe AKI) if it was stage 2 or 3 [21]. Baseline creatinine was estimated using a previously validated height-independent approach assuming a GFR of 120 mL/min per 1.73m^2^ as previously described [22]. The eGFR value was adjusted for the difference between estimated and measured GFR values by age using iohexol clearance. Adding a constant value of 16 to age-based norms across age groups was used to account for creatinine-based over-estimation of GFR [23]. Creatinine was tested using the modified Jaffe colorimetric method on an Alinity c instrument (Abbott, Lake Forest, IL) which is traceable to an isotope dilution mass spectrometry (IDMS) reference method.

### Measurement of biomarkers of host response to infection

Biomarkers of immune and endothelial activation were evaluated in EDTA anticoagulated plasma stored at − 80 °C using a custom Magnetic Luminex® Performance Assay (R&D Systems) [19, 24]. The markers assessed included Cystatin C, C-X-C motif chemokine Ligand 10 (CXCL10) / interferon γ-induced protein 10 kDa (IP-10), chitinase-3-like protein 1 (CHI3L1), soluble tumor necrosis factor receptor-1 (sTNFR1), soluble triggering receptor expressed on myelocytes (sTREM-1), interleukin 6 (IL-6), and interleukin 8 (IL-8) (representing immune activation), as well as angiopoietin-2 (Angpt-2), angiopoietin-1 (Angpt-1), soluble fms-like tyrosine kinase-1 (sFlt-1), soluble vascular cell adhesion molecules (VCAM-1), and soluble intercellular adhesion molecule-1 (sICAM-1) (representing endothelial activation).

### Statistical analysis

Data were analyzed using STATA/SE v17.0 and GraphPad Prism v7.0. Continuous data were analyzed using Student’s *t*-test or Wilcoxon rank-sum test. Categorical data were analyzed using Pearson’s Chi-square test. The frequency of missing data is reported in Table 1. In instances where continuous data were missing and included in subsequent regression models (e.g., length), data were imputed using the participant age and weight. Logistic regression was used to evaluate the association between BWF and severe AKI adjusting for potential confounders. Poisson regression with robust variance estimation was used to evaluate the association between severe AKI and BWF and mortality. To evaluate the relationship between BWF and severe AKI and mortality, we used multivariable logistic regression models adjusting for age, sex, and a composite measure of disease severity with the Lambaréné Organ Dysfunction Score (LODS) [18, 25]. Poisson regression was used to define the association between biomarkers of immune and endothelial activation and severe AKI and BWF, with log_e_ transformation of biomarkers and adjusting for age and sex. To evaluate relative differences in biomarker signatures across groups, biomarker concentrations were standardized to have a mean of 0 and standard deviation of 1 within the sub-study population. The mean standardized concentrations are presented by severe AKI and BWF status. Differences in biomarker concentrations were evaluated using the standardized biomarkers as the dependent variable and severe AKI and BWF in a factorial model design. The reference category was children without severe AKI or BWF and differences in severe AKI and BWF and the interaction were evaluated. To account for multiple comparisons, the Bonferroni method was used as indicated.

**Table 1:** Characteristics of study population based on a history of blackwater fever

	***N***	**Cohort (*n* = 999)**	**No BWF** **(*n* = 917)**	**BWF** **(*n* = 82)**	***P* value**
**Demographics**					
Age, years	996	1.67 (1.06)	1.64 (1.06)	1.92 (1.05)	0.022
Male sex, *n* (%)	989	550 (55.6)	499 (54.8)	51 (64.6)	0.095
Weight, kg	995	9.8 (3.1)	9.8 (3.2)	10.7 (2.5)	0.009
Length, cm	979	74.1 (11.6)	73.9 (11.7)	77.1 (10.8)	0.015
**Infection status**					
RDT result, n (%)					
Negative	999	280 (28.0)	264 (28.8)	16 (19.5)	0.053
HRP-2 alone		143 (14.3)	123 (13.4)	20 (24.4)	
HRP-2/LDH positive		534 (53.5)	492 (53.7)	42 (51.2)	
pLDH alone		9 (0.9)	8 (0.9)	1 (1.2)	
Missing		33 (3.3)	30 (3.3)	3 (3.7)	
HIV, *n* (%)	998	21 (2.1)	20 (2.2)	1 (1.2)	1.000
**Medication history**					
Antimalarial, *n* (%)	989	458 (46.4)	422 (46.6)	36 (43.9)	0.642
Antibiotic, *n* (%)	987	337 (34.2)	308 (34.1)	29 (35.4)	0.813
**Clinical signs and symptoms**					
Axillary temperature in °C	986	37.9 (1.2)	37.9 (1.2)	37.5 (1.2)	0.003
Systolic blood pressure, mmHg	962	105 (16)	105 (16)	108 (14)	0.055
Diastolic blood pressure, mmHg	960	58 (13)	58 (13)	57 (13)	0.198
Heart rate, bpm	989	160 (25)	160 (25)	158 (24)	0.535
Respiratory rate, bpm	959	45 (15)	45 (14)	47 (15)	0.258
Capillary refill time > 2 s, *n* (%)	972	133 (13.7)	111 (12.4)	22 (28.6)	< 0.0001
Unable to drink or breastfeed, *n* (%)	993	189 (19.0)	158 (17.3)	31 (37.8)	< 0.0001
Vomiting, *n* (%)	995	306 (30.8)	267 (29.2)	39 (48.2)	< 0.0001
Diarrhea, *n* (%)	996	299 (30.0)	271 (29.7)	28 (34.2)	0.395
Respiratory distress^a^, *n* (%)	999	316 (31.6)	280 (30.5)	36 (43.9)	0.013
Prostration, *n* (%)	996	227 (22.8)	192 (21.0)	35 (42.7)	< 0.0001
Altered consciousness, *n* (%)	993	138 (13.9)	115 (12.6)	23 (28.1)	< 0.0001
Convulsions, *n* (%)	998	176 (17.6)	159 (17.4)	17 (20.7)	0.443
Severe anemia (Hb < 5.0 g/dL), *n* (%)					
No	999	187 (18.7)	181 (19.7)	6 (7.3)	< 0.0001
Yes		207 (20.7)	167 (18.2)	40 (48.8)	
Missing		605 (60.6)	569 (62.1)	36 (43.9)	
Jaundice, *n* (%)	998	110 (11.0)	74 (8.1)	36 (43.9)	< 0.0001
**Disease severity**					
LODS^b^, *n* (%)					
0	997	635 (63.7)	598 (65.3)	37 (45.7)	< 0.0001
1		187 (18.8)	169 (18.5)	18 (22.2)	
2		110 (11.0)	101 (11.0)	9 (11.1)	
3		65 (6.5)	48 (5.2)	17 (21.0)	

Data presented as mean (SD) or *n* (%)

^a^Defined based on the presence of nasal flaring, deep breathing, or intercostal/subcostal retractions

^b^Lambaréné Organ Dysfunction Score

### Role of the funding source

The funders had no role in the study design, analysis, or decision to publish.

## Results

**Fig. 1:**
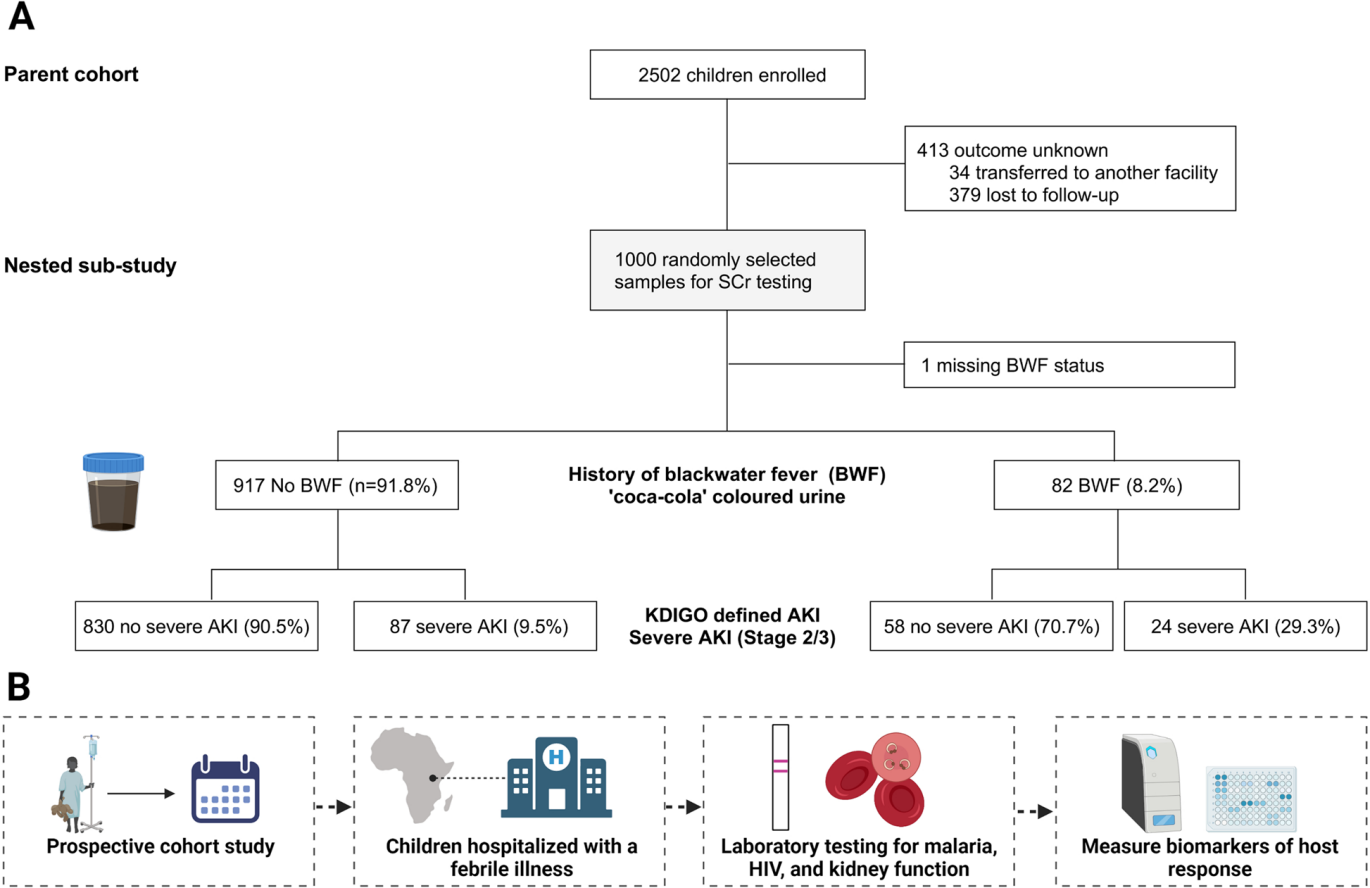
Flow chart of study population. **A** Flow chart of study population. **B** Overview of study activities

Kidney function was assessed in 999 randomly selected children from among 2502 children enrolled in the parent study with BWF and AKI status known (Fig. 1). The mean (SD) age at admission was 1.66 years (1.06) and 55.7% of participants were male. Malaria was diagnosed in 687 participants (71.0%). Antimicrobial pretreatment was common with 34.2% of children reporting antibiotic use and 46.4% reporting antimalarial use (Table 1). Children who were RDT positive for HRP-2 alone were most likely to report antimalarial pretreatment (57.0%) followed by children who were malaria negative (52.2%). Children who were malaria positive were least likely to report antimalarial use (40.5%). The etiology of febrile illness among malaria-negative children was unknown as blood cultures and other microbiological investigations were not available on site.

### Frequency and risk factors for BWF at presentation

On admission, 82 children (8.2%) had a history of BWF. Children with BWF were more likely to be male and older than children without BWF (Table 1). Children with BWF were more likely to present with hypovolemia (delayed capillary refill time, an inability to drink or breastfeed, vomiting) and had signs of greater disease severity with an increased frequency of prostration, respiratory distress, coma, severe anemia, and jaundice compared to children without BWF (Table 1). Although there was no difference in BWF based on the presence or absence of malaria by RDT, children with BWF were more likely to test positive for HRP-2 alone.

### Frequency and risk factors for AKI at presentation

The prevalence of AKI on admission was 49.5% with 11.1% of children having severe AKI defined as stage 2 or 3 AKI. Among children with AKI, 77.6% were stage 1, 16.4% stage 2, and 6.1% stage 3. Characteristics associated with severe AKI are presented in Table 2. Children with severe AKI were older, were more likely to be male, have clinical signs of hypovolemia (delayed capillary refill time, history of vomiting and unable to drink), and have greater illness severity and multi-organ dysfunction (impaired consciousness, prostration, respiratory distress, jaundice, severe anemia, and BWF) (*p* < 0.05 for all).

**Table 2:** Characteristics associated with severe AKI on hospital admission

	**No AKI** **(*n* = 888)**	**Severe AKI** **(*n* = 111)**	***P* value**
**Demographics**			
Age, years	1.64 (1.07)	1.87 (0.98)	0.038
Male sex, *n* (%)	446 (54.1)	74 (67.9)	0.006
Weight, kg	9.8 (3.1)	10.5 (3.0)	0.017
Length, cm	73.7 (11.5)	78.1 (11.9)	0.0002
**Medication history**			
Antimalarial, *n* (%)	404 (46.0)	54 (49.5)	0.480
Antibiotic, *n* (%)	295 (33.6)	42 (38.5)	0.310
**Infection status**			
Malaria infection, *n* (%)			
Negative	246 (27.7)	34 (30.6)	0.284
HRP-2 positive alone	122 (13.7)	21 (18.9)	
pLDH and HRP-2 positive	483 (54.4)	51 (46.0)	
pLDH positive alone	9 (1.0)	0 (0.0)	
Missing	28 (3.2)	5 (4.5)	
HIV, *n* (%)	17 (1.9)	4 (3.6)	0.279
**Clinical signs and symptoms**			
Axillary temperature in °C	37.9 (1.2)	37.6 (1.2)	0.029
Systolic blood pressure, mmHg	105 (16)	104 (15)	0.651
Diastolic blood pressure, mmHg	58 (13)	55 (12)	0.073
Heart rate, bpm	160 (25)	156 (25)	0.088
Respiratory rate, bpm	45 (14)	45 (15)	0.718
Capillary refill time > 2 s, *n* (%)	110 (12.7)	23 (21.5)	0.013
Unable to drink or breastfeed, *n* (%)	155 (17.6)	34 (30.9)	0.001
Vomiting, *n* (%)	253 (28.6)	53 (48.2)	< 0.0001
Diarrhea, *n* (%)	267 (30.1)	32 (29.1)	0.822
Respiratory distress^a^, *n* (%)	268 (30.2)	48 (43.2)	0.005
Prostration, *n* (%)	178 (20.1)	49 (45.0)	< 0.0001
Coma (BCS < 3) *n* (%)	35 (4.0)	16 (15.1)	< 0.0001
Altered consciousness, *n* (%)	105 (11.9)	33 (30.0)	< 0.0001
Convulsions, *n* (%)	158 (17.8)	18 (16.2)	0.677
Jaundice, *n* (%)	79 (8.9)	31 (27.9)	< 0.0001
Severe anemia (Hb < 5.0 g/dL), *n* (%)			
No	164 (18.5)	23 (20.7)	0.002
Yes	171 (19.2)	36 (32.4)	
Missing	553 (62.3)	52 (46.9)	
Blackwater fever, *n* (%)	58 (6.5)	24 (21.6)	< 0.0001
**Disease severity**			
LODS^b^, *n* (%)			
0	584 (65.8)	51 (46.4)	< 0.0001
1	168 (18.9)	19 (17.3)	
2	93 (10.5)	17 (15.5)	
3	42 (4.7)	23 (20.9)	

Data presented as mean (SD) or *n* (%) unless otherwise stated

^a^Defined based on the presence of nasal flaring, deep breathing, or intercostal/subcostal retractions

^b^Lambaréné Organ Dysfunction Score

### Association between BWF and AKI

The rate of severe AKI among patients with BWF was 29.3%, compared to 9.5% in children without BWF (*p* < 0.001) (Fig. 1). To evaluate whether BWF represents an independent risk factor for severe AKI, we conducted multivariable logistic regression adjusting for age, sex, length, respiratory distress, altered consciousness, jaundice, severe anemia, and signs of hypovolemia (vomiting, delayed capillary refill time, or being unable to drink or breastfeed). A history of BWF on admission was an independent predictor of severe AKI and was associated with a 2.18-fold increase in the odds of severe AKI (95% CI 1.15 to 4.16). We further explored the relationship between sex, BWF, and severe AKI by including an interaction term between BWF and sex in the multivariable logistic regression model. Among males with BWF, there was a 4.21-fold (95% CI 1.92, 9.24) increase in the odds of severe AKI (*p* < 0.0001). Neither females with BWF nor males without BWF had an increased risk of severe AKI.

### Relationship between AKI and BWF and immune and endothelial activation

**Fig. 2:**
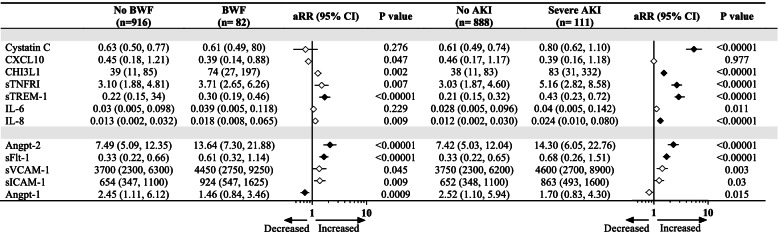
Relationship between host markers of inflammation and endothelial injury, blackwater fever, and severe AKI. Forest plot depicting the median (interquartile range) of biomarkers of immune and endothelial activation by blackwater fever (BWF) and severe AKI status with the corresponding adjusted risk ratio (aRR) and 95% confidence interval (95% CI) generated from a Poisson model with robust variance adjusting for participant age and sex. Biomarkers significant following adjustment for multiple comparisons (*n* = 24) using the Bonferroni method are shaded in black with an adjusted *p* < 0.002

To begin to elucidate pathways underlying severe AKI and BWF, we quantified biomarkers involved in the host response to infection (Fig. 2). Children with severe AKI had elevated Cystatin C as well as markers of immune (CHI3L1, sTNFR1, sTREM-1, and IL-8) and endothelial (Angpt-2, sFlt-1) activation compared to children without severe AKI (adjusted *p* < 0.05). Children with BWF had increased endothelial activation with increases in Angpt-2 and sFlt-1 and decreases in Angpt-1 (adjusted *p* < 0.05). However, fewer markers of immune activation were deranged, with only sTREM-1 being statistically significantly elevated after adjustment for multiple comparisons (*p* < 0.0001).

**Fig. 3:**
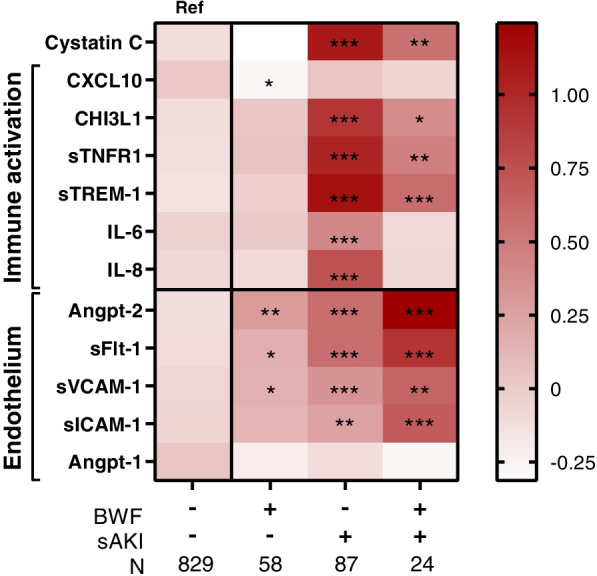
Blackwater fever and severe AKI in children with an acute febrile illness. Heat map of standardized biomarker levels with the mean in each group plotted and differences between groups analyzed using linear regression models. Biomarkers are the dependent variable and severe AKI (sAKI) and blackwater fever (BWF) are predictor variables, with an interaction term, where *p < 0.05, **p < 0.01, ***p < 0.001

To evaluate potential interactions between severe AKI and BWF-mediated immune and endothelial activation, patterns in standardized biomarker levels across groups were studied (Fig. 3). Consistent with the regression analysis (Fig. 2), the presence of severe AKI alone was associated with higher concentrations of markers of immune and endothelial activation compared to the reference group of children without BWF or severe AKI (Fig. 3). BWF alone was associated with higher Angpt-2, sFlt-1, and sICAM-1 compared to the reference group of children without BWF or severe AKI. Children with both severe AKI and BWF had higher inflammation (CHI3L1, sTNFR1, and sTREM-1) compared to children without severe AKI or BWF, but less inflammation compared to children with severe AKI alone (77.6%). Children with both severe AKI and BWF had evidence of enhanced endothelial activation compared to children with severe AKI or BWF alone.

### AKI, not BWF, is associated with mortality

**Fig. 4:**
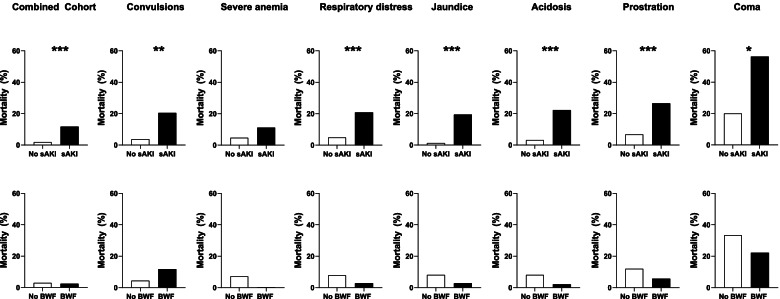
Mortality in hospitalized children across different clinical complications based on the presence of severe AKI or blackwater fever (BWF). Bar graphs depicting the mortality rate in children with severe AKI (stage 2 or 3 AKI, sAKI) or a history of blackwater fever (BWF) based on clinical complications present on admission. Mortality was assessed based on sAKI or BWF using Pearson’s Chi-square or Fisher’s exact test and relationships significant (*p* < 0.05) are depicted with an *

The overall mortality was 2.9%. Death occurred in 4.2% of children with AKI compared to 1.6% of children without AKI (*p* = 0.012). Mortality was higher in children with severe AKI (11.7%) compared to children without severe AKI (1.8%) (*p* < 0.001) corresponding to a 2.17 increased risk ratio for mortality with severe AKI (95% CI 1.01 to 4.64), adjusting for age and sex and disease severity. In-hospital mortality was 2.4% among children with BWF compared to 3.0% among children without BWF (*p* = 0.793). Adjusting for age, sex, and disease severity, a history of BWF was associated with a reduced risk of in-hospital mortality (RR, 0.26 95% CI 0.07 to 0.996). To further explore whether the relationship between AKI and BWF and mortality was affected by clinical complications at presentation, we conducted additional analyses evaluating mortality in children across different clinical complications (Fig. 4). The presence of severe AKI was associated with a significant increase in mortality in children presenting with coma, prostration, acidosis, jaundice, respiratory distress, and convulsions (*p* < 0.05), while BWF was not associated with mortality across any subgroup.

## Discussion

In the present study, 8.2% of children had a history of BWF and nearly 50% of children hospitalized with an acute febrile illness presented with AKI. BWF was independently associated with severe AKI with the greatest risk occurring in males. AKI was associated with high inflammation and endothelial activation, whereas children with BWF had endothelial activation without accompanying immune activation. Severe AKI was an independent risk factor for mortality while children with BWF may have a lower risk of in-hospital death.

In this study, the overall prevalence of BWF was 8.2%, which is consistent with prevalence estimates from the region. A large multi-site study identified BWF in 12.4% of hospitalized children with severe febrile illness (range: 0.5 to 21.8%) across several sites in East Africa [11]. Sites in Uganda that were geographically closest to the study site reported a BWF prevalence of 6.2% and 14.5% [11]. A subsequent study conducted at the same hospital as the present study reported BWF in 33% of 279 children hospitalized with severe anemia [17]. Among the 394 children with hemoglobin assessed in this study, 19.3% of children with severe anemia had BWF.

Consistent with previous reports, children who were RDT positive for HRP-2 alone (suggestive of recent malaria infection) had a higher frequency of BWF compared to children without malaria [11]. It has been speculated that exposure to antimalarial drugs, in particular artemisinin derivatives, may be associated with an increased risk of hemolysis [11]. To date, population-based studies in African children with malaria have not demonstrated an increased risk of delayed hemolysis following treatment with artemisinin-derivatives [26, 27] but there have been isolated reports of BWF following severe malaria treatment [28]. In the present study, children who reported recent antimalarial use were not at increased risk of BWF or AKI.

There was a strong relationship between BWF and severe AKI in this study which is consistent with BWF as a risk factor for severe AKI and kidney failure [29, 30]. However, due to the cross-sectional nature of the study design, it is difficult to ascertain the temporal nature of the relationship. The relationship between BWF and AKI was strongest in males and consistent with male sex as a risk factor for BWF in some [11], but not all [17], cohorts. We speculate this may be related to G6PD deficiency, an inherited red blood cell disorder on the X chromosome which is a highly polymorphic gene and deficiencies are more common among hemizygote males. In Ugandan children, deficiency in three G6PD allelic variants were not associated with a marked increase in BWF [11, 31]; however, among Vietnamese adults with malaria, novel G6PD allelic variants identified by sequencing were associated with BWF [32]. It is possible that regional or sub-regional differences in G6PD allelic variants may contribute to increased BWF and severe AKI observed in Eastern Uganda, and sex-based differences in susceptibility [11, 17, 33]. We hypothesize that intravascular hemolysis resulting in cell-free hemoglobin levels exceeding the buffering capacity of hemoglobin scavengers will contribute to the development of BWF and predispose children to AKI.

To our knowledge, this is the first study to assess pathways of immune and endothelial activation in the context of AKI and BWF. Although there was overlap in BWF and AKI risk factors, consistent with hemolysis-mediated endogenous nephrotoxins being risk factors for AKI (e.g., anemia, jaundice) [9], there were distinct differences in patterns of the host biologic response. Severe AKI was associated with higher immune and endothelial activation, including previously identified AKI biomarkers (CHI3L1, sTREM-1, sTNFR1, Angpt-2) [34–38]. In contrast, children with BWF had systemic endothelial activation without a corresponding increase in immune activation. Additional studies are needed determine whether BWF dampens immune activation in the context of severe AKI, or whether BWF may be an alarming symptom prompting parents to bring their children to hospital earlier in illness before a robust inflammatory response is initiated. Prospective studies are needed to further evaluate the relationship between hemolysis and AKI.

Mortality in this study (2.9%) was lower than another large study of febrile children with impaired perfusion in Africa (~ 7%) [39], as well as controlled trials of children with severe malaria (~ 8%) [40] and pneumonia (12–15%) [41]. Because our study enrolled a consecutive sample of all hospitalized febrile children, the cohort includes a full and representative range of moderate and severe febrile illness, accounting for the lower mortality compared to studies of shock, severe malaria, and hypoxemic pneumonia. The association of AKI with mortality in our study of febrile children is similar to previous studies in children with malaria [5, 6, 42]. Of note, a large proportion (71%) of our cohort had malaria. Among 331 children hospitalized in Nigeria, AKI occurred in 33.8% of children and the presence of AKI was associated with a 2.9-fold increase in overall mortality with sepsis (33%) and malaria (13%) the primary diagnoses [43].

Strengths of this study include the large sample size which enabled investigation of the inter-relationships between AKI and BWF among children hospitalized with an acute febrile illness. The assessment of biomarkers associated with life-threatening responses to infection allowed us to investigate differences in host response in children with AKI and BWF and identify unique “bio-signatures” that are more amenable to intervention. While children with BWF were more likely to have severe AKI, they had lower in-hospital mortality. However, BWF is a risk factor for hospital readmission, post-discharge mortality, and the presence of persistent kidney disease at 1-month follow-up [16, 17, 33]. Together, these data suggest that children with BWF are a high-risk population requiring clinical follow-up to assess kidney recovery and identify risk factors for ongoing hemolysis.

Limitations in this study include the lack of data on urine output to define AKI, and urinalysis to confirm the diagnosis and to speculate on the etiology of BWF fever and AKI. AKI was defined based on a single creatinine measurement using the Modified Jaffe method which is susceptible to interference and may have led to misclassification of AKI [44]. For this reason, many of our analyses were restricted to severe AKI. While the use of more specific enzymatic methods to measure creatinine are preferable, particularly in children, barriers in access and cost precluded the use of enzymatic methods. Additional longitudinal studies are needed to define the kinetics of immune and endothelial activation in children with BWF using longitudinal sampling. Diagnosis of malaria by RDT was a limitation that may have missed *Plasmodium malariae* infection [45], which is a risk factor for nephropathy [46]. In addition, we cannot comment on the etiology of non-malarial febrile illness within the cohort. An important limitation in extending our understanding between hemolysis and blackwater fever and AKI is the incomplete data on hemoglobin for the cohort and our inability to measure cell-free hemoglobin and heme as markers of hemolysis. Finally, as this study only followed children until hospital discharge, we could not assess the relationship between AKI and BWF and post-discharge morbidity and mortality. Additional studies are needed to understand the relationship between host genetics, hemolysis, and AKI in malaria-endemic areas, with an emphasis on pediatric populations in Africa.

## Conclusions

AKI and BWF are related syndromes in children with severe febrile illnesses, and are associated with malaria, severe anemia, and jaundice. Together, the findings suggest that hemolysis contributes to AKI in the African setting. Increased awareness of AKI in the setting of BWF and improved access to AKI diagnostics is needed to reduce disease progression and in-hospital mortality in this high-risk group of children through early implementation of kidney-protective measures.

## Data Availability

The datasets used and/or analyzed during the current study are available from the senior author on reasonable request (email: kevin.kain@uhn.ca).

## Abbreviations

AKI: Acute kidney injury
Angpt: Angiopoietin
BWF: Blackwater fever
CXCL10: C-X-C motif chemokine Ligand 10
CHI3L1: Chitinase-3-like protein 1
Flt-1: Fms-like tyrosine kinase-1
HRP-2: Histidine Rich Protein-2
ICAM-1: Intercellular adhesion molecule-1
IP-10: Interferon γ-induced protein 10 kDa
IL-6: Interleukin 6
IL-8: Interleukin 8
IDMS: Isotype dilution mass spectrometry
LODS: Lambaréné Organ Dysfunction Score
LMIC: Low-and-middle-income countries
pLDH: Pan-malaria lactate dehydrogenase
RDT: Rapid Diagnostic Test
TNFR1: Tumor necrosis factor receptor-1
TREM-1: Triggering receptor expressed on myelocytes
VCAM-1: Vascular cell adhesion molecules
